# Analysis of TATA-box binding protein associated factor 4b gene mutations in a Chinese population with nonobstructive azoospermia

**DOI:** 10.1097/MD.0000000000020561

**Published:** 2020-06-05

**Authors:** Qi Xi, Hao Zhang, Xinyue Zhang, Yuting Jiang, Ruixue Wang, Ruizhi Liu, Hongguo Zhang

**Affiliations:** a Center for Reproductive Medicine and Center for Prenatal Diagnosis, First Hospital, Jilin University, Changchun; b Center for Reproductive Medicine, Yanbian University Hospital, Yanji, China.

**Keywords:** azoospermia, infertility, male, polymorphism, single nucleotide

## Abstract

Nonobstructive azoospermia (NOA) is a severe form of male infertility. The molecular basis of NOA is still poorly understood. The aim of this study was to explore the associations between single nucleotide polymorphisms (SNPs) of the TATA-box binding protein associated factor 4b (*TAF4B*) gene and NOA. A total of 100 Han Chinese patients with NOA and 100 healthy men as controls were recruited. Targeted gene capture sequencing was performed. A total of 11 TAF4B SNPs were screened in the NOA and control subjects. Six synonymous and 4 nonsynonymous variants were detected. The c.11G>T (p.G4V) mutation was detected only in NOA patients. Polymorphism Phenotyping v2 and Sorting Intolerant From Tolerant analysis indicated that the p.G4V mutation influenced the protein structure of TAF4B. Haplotype analysis showed that the candidate SNPs did not independently associate with NOA and were found at extremely low frequencies in the subject population. Mutation Taster analysis indicated that the c.11G>T/p.G4V mutation was damaging. WebLogo analysis showed that the residue at amino acid 4 was relatively conserved. The p.Gly4Val substitution may affect the structure of the TAF4B protein. The c.11G>T mutation of the *TAF4B* gene may be associated with NOA in a Chinese population. Bioinformatics analysis indicated this variation may play an important role in the process of spermatogenesis.

## 1 Introduction

Male infertility is a multifactorial reproductive health problem. Most male infertility is caused by the absence of spermatozoa in the testes (azoospermia) or distinct alterations of sperm quality.^[1]^ Nonobstructive azoospermia (NOA) affects 60% of men with azoospermia.^[2]^ Until now, the etiology of NOA, especially the detailed molecular mechanisms, has remained largely unknown. In the past 10 years, the genetic tests become widespread for the definite etiology. More than 2000 genes are involved in spermatogenesis,^[3]^ and mouse models have identified over 400 genes specifically linked to azoospermia. Although increasing numbers of genes associated with NOA have been reported through case reports and mouse model studies, the molecular basis of NOA is still poorly understood.^[4]^ Only a small number of genes associated with azoospermia proposed by mouse models have been identified in humans, such as testis expressed 11 (*TEX11),* tudor domain containing 9 (*TDRD9),* Zinc finger MYND-type containing 15 (*ZMYND15)*, and TATA-box binding protein associated factor 4b (*TAF4B*).^[5]^

TAF4B is a cell type-specific TBP-associated factor that may mediate transcription by a subset of activators in B cells. *TAF4B* is located on human chromosome 18q11.1 and is highly expressed in testis (RPKM 5.0), lymph node (RPKM 3.4), and 24 other tissues.^[6]^ In mouse, TAF4B is a gonadal-enriched component of the general transcription factor complex, transcription factors IID (TFIID), which is required for the maintenance of spermatogenesis.^[7]^*TAF4B* expression may affect the development of spermatogenic cells spermatogonial stem cells.

In the present study, we performed targeted gene capture sequencing to identify mutations of *TAF4B* among 100 patients with NOA and 100 controls. Bioinformatic analysis combined with case-control studies was conducted to systematically assess the effects of mutations on TAF4B structure.

## 2 Material and methods

### 2.1 Study population

A total of 100 Han Chinese patients with NOA (age 29.14 ± 4.40 years) and 100 healthy men as a control group (age 25.10 ± 5.68 years) were recruited from the Center for Reproductive Medicine, the First Hospital of Jilin University. Patients with abnormal karyotypes and Y chromosome microdeletions were excluded from the study. Controls were healthy men randomly chosen with a normal sperm count and no known history of infertility. This study was approved by the ethics committee of the First Hospital of Jilin University and all patients gave written informed consent.

### 2.2 Sequencing and mutational analysis

Genomic deoxyribonucleic acid was isolated from blood lymphocyte samples. Biotinylated capture probes were designed for *TAF4B* gene exons and mutation screening of genes was performed by targeted gene capture sequencing using the Illumina HiSeq2000 Next-Generation Sequencing platform (MyGenostics, Beijing, China) according to our previously published paper.^[8]^ The impact of the mutations on TAF4B protein was assessed by Polymorphism Phenotyping v2 (PolyPhen-2) and Sorting Intolerant From Tolerant (SIFT). Detailed mutation information was predicted by MutationTaster (http://www.mutationtaster.org/). Statistical analysis was performed with SPSS Inc., version 19.0 (IBM Corp. Armonk, NY, USA) and *P* < .05 was considered statistically significant.

### 2.3 Haplotype and conservation analysis

Haplotype analysis was performed with Haploview 4.2. Haploview was used to generate linkage disequilibrium blocks to determine which of the 11 SNPs were inherited together as haplotypes. Multiple species amino acid sequence alignment and WebLogo analysis of the TAF4B protein were carried out separately by European Molecular Biology Laboratory-European Bioinformatics Institute (EMBL-EBI) (https://www.ebi.ac.uk/Tools/msa/muscle/) and WebLogo Version 2.8.2.^[9]^

### 2.4 Structural analysis

Structural analysis of the *TAF4B* variant was performed using SWISS-MODEL software (https://www.swissmodel.expasy.org/; based on the template of the Transcription initiation factor TFIID subunit 4, 2p6v.pdb) and Protein Fold recognition Server (Phyre2). For protein structure visualization, we used PyMol Version 2.2.0.

## 3 Results

**Figure 1:**
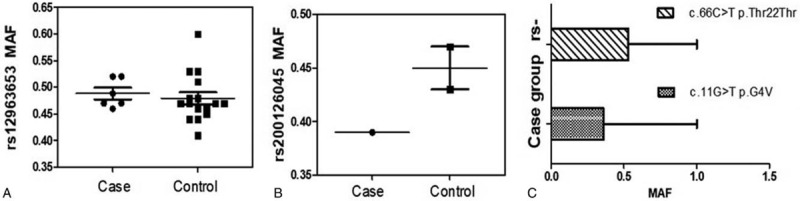
MAF analysis of c.1616A>G, c.1619A>C, and c.11G>T. MAF = minimum allele frequency.

**Figure 2:**
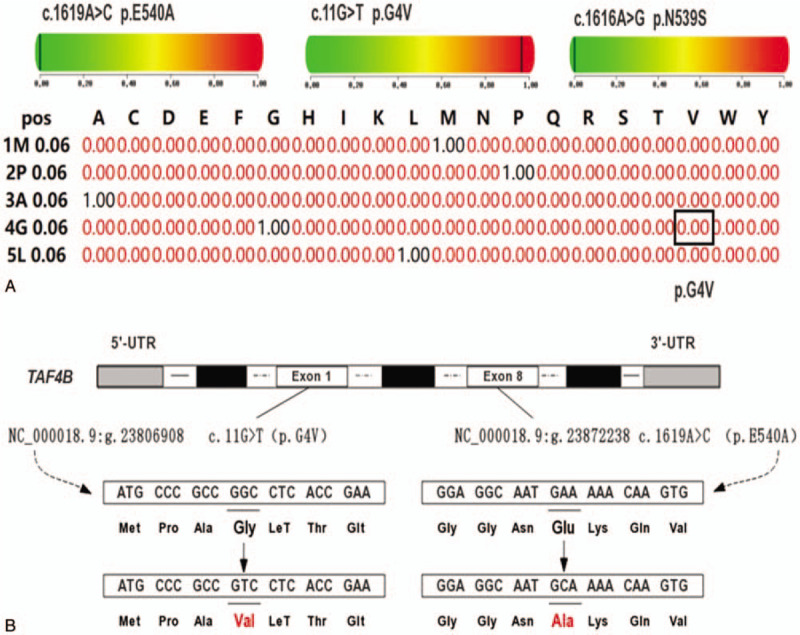
(A) PolyPhen-2 and SIFT analysis of p.G4V mutation; (B) location of mutations (c.11G>T and c.1619A>C) in the *TAF4B* gene. SIFT = sorting intolerant from Tolerant, TAF4B = TATA-box binding protein associated factor 4b.

Targeted gene capture sequencing of *TAF4B* in 100 NOA patients and 100 controls identified 6 synonymous variants (rs12456749, rs1677016, rs17224558, rs3744961, rs3826624, and rs771186391) and 4 nonsynonymous variants (rs12963653, rs148172329, rs200126045, and rs74947492) (Table 1). SIFT showed that p.G492G, p.N539S, p.Q375H, p.E540A, p.V438L, and p.G4V mutations were tolerable. The p.G4V mutation was only detected in NOA patients and not in the controls. For the sites with significant differences in nonsynonymous mutations, the minimum allele frequency of the mutations is shown in Figure 1. Figure 1 also shows the minimum allele frequency of the novel mutation (c.11G>T/p.G4V) that was detected only in NOA patients. The PolyPhen-2 and SIFT analysis indicated that the p.G4V mutation influenced the protein structure and function (SIFT sensitivity: 0.99, specificity: 0.14; PolyPhen-2 intersection points: 0.00) (Fig. 2A). Bioinformatics analysis indicated that c.11G>T led to an amino acid substitution at the fourth residue (p.G4V) and c.1619A>C led to an amino acid substitution at 540th residue (p.E540A) (Fig. 2B).

**Table 1:**
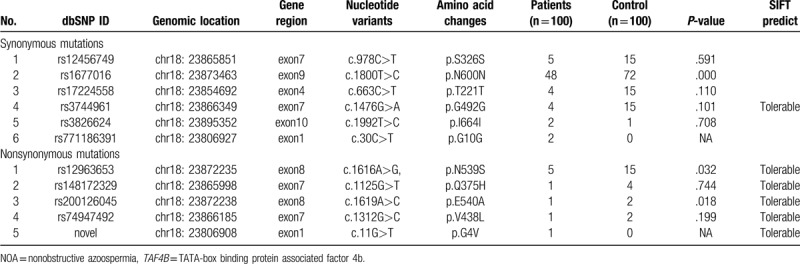
TAF4B mutations identified in NOA patients and controls.

**Figure 3:**
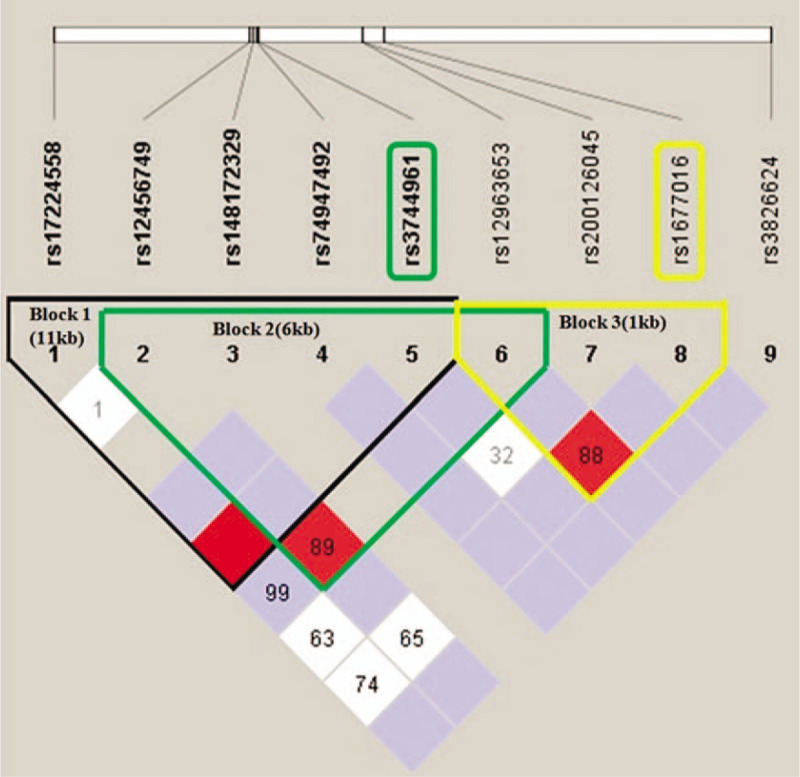
Haplotype analysis of NOA-SNPs on chromosome 18. NOA = nonobstructive azoospermia, SNPs = single nucleotide polymorphisms.

The 11 candidate SNPs were distributed on chromosome 18. Two linkage disequilibrium blocks were identified within rs3744961 (Fig. 3, black and green box), and 1 linkage disequilibrium block was identified within rs1677016 (Fig. 3, yellow box) of *TAF4B*. However, haplotype analysis showed that the candidate SNPs did not independently associate with NOA and created haplotypes that were found at extremely low frequencies in the subject population.

MutationTaster programs were used to predict the impact of disease-causing variants on protein structure, function, and disease-causing potential of sequence variations. MutationTaster analysis indicated that the c.11G>T/p.G4V mutation was damaging and this mutation is not present in the dbSNP, 1000 Genome, ExAC, and gnomAD databases.

**Figure 4:**
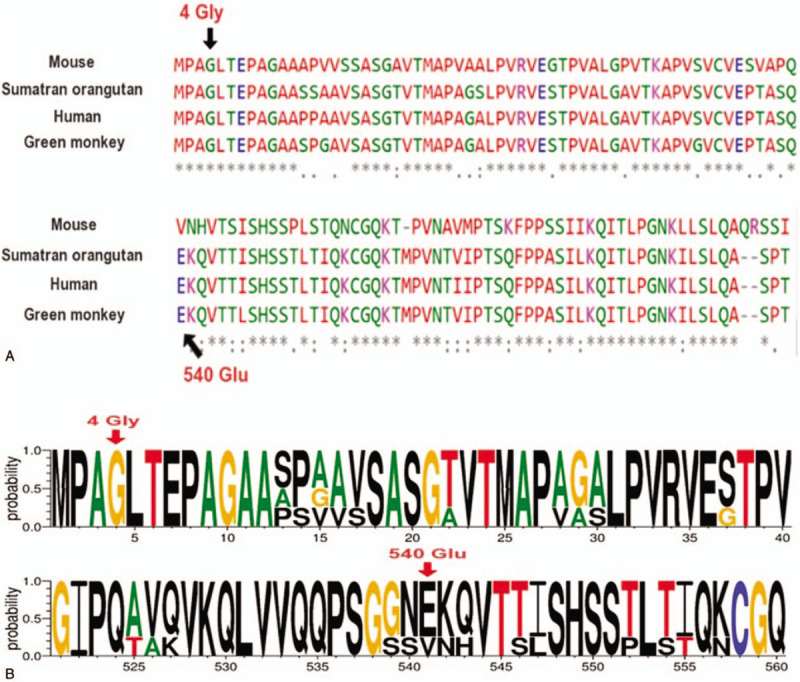
(A) Evolutionary conservation analysis of multiple sequence alignments of TAF4B protein; (B) WebLogo analysis showed that the residue at amino acid 4 was relatively conserved. TAF4B = TATA-box binding protein associated factor 4b.

Evolutionary conservation analysis of multiple sequence alignments of TAF4B protein and its homologs showed that the glycine at residue 4 was evolutionarily conserved from the green monkey to human (Fig. 4A), suggesting that Gly4 may play an important role in the function of the TAF4B protein. WebLogo analysis showed that the domain was relatively conserved from the green monkey to humans (Fig. 4B).

**Figure 5:**
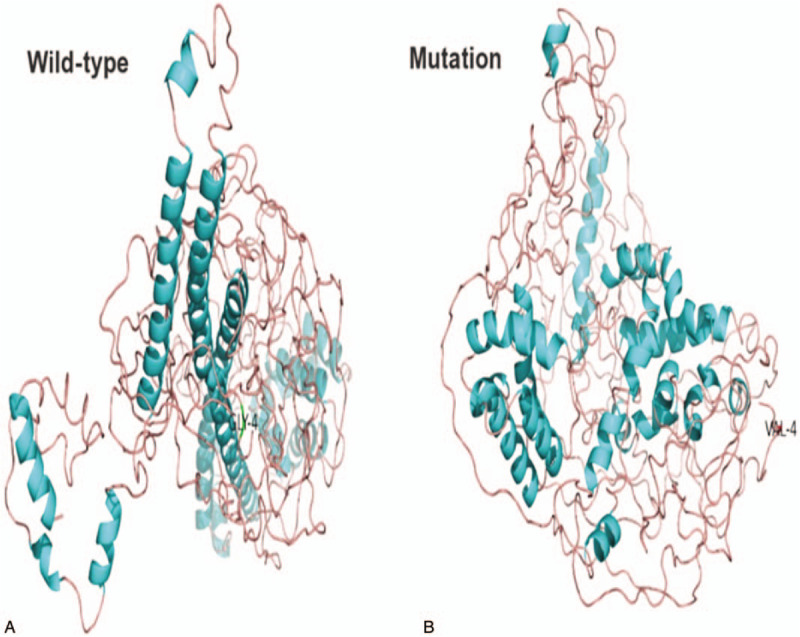
Structural analysis of the wild-type and mutant TAF4B proteins (c.11G>T/p.G4V) predicted that the protein folding structure changed in the mutant protein. TAF4B = TATA-box binding protein associated factor 4b.

Structural analysis of the wild-type and mutant TAF4B proteins (c.11G>T/p.G4V) predicted that the protein folding structure was altered in the mutant protein (Fig. 5). The folder structure of the protein breaks when the amino acid residue 4 changes, which suggests that the p. Gly4Val substitution may affect the structure of the TAF4B protein.

## 4 Discussion

Approximately 50% of infertility cases are associated with male factors, and azoospermia is prevalent in 5% of infertile men.^[5]^ For patients with NOA, the etiology is mostly idiopathic and only a minority of cases carry a defective karyotype or a Y-chromosome microdeletion. The underlying etiology and genetic mechanism of NOA remain largely unclear. Current research has shown that genetic defects are the main causes of abnormal spermatogenesis, especially for NOA.^[10–13]^ Hence, discovering the underlying etiology of NOA is important. A massively parallel sequencing was performed to identify genetic abnormalities in a large cohort including NOA patients and controls.^[14]^ Such studies can help researchers explore the underlying genetic aetiologies of NOA. In this study, we aimed to identify and investigate the genetic mutations of the *TAF4B* gene in a Chinese population with NOA. A nonsynonymous mutation in exon 1 of the *TAF4B* gene (c.11G>T/p.G4V) was identified in NOA patients and not detected in 100 healthy men. Our analysis indicates that this mutation may have an irreversible effect on the TAF4B protein. Therefore, this study focused on the application of bioinformatics analysis to explore whether the c.11G>T mutation of *TAF4B* gene is related to the occurrence of NOA.

The gene encoding TAF4B, also called TAFII105 (RNA polymerase II, TATA box-binding protein-associated factor), has 15 exons and encodes an 862 amino acid protein.^[15]^ The TFIID complex is a core RNA polymerase complex that contains the TATA-binding protein and 14 TBP-associated factors that function in core promoter recognition and activator-dependent RNA polymerase II recruitment.^[16]^ Freiman et al^[17]^ reported that TAF4B is enriched in mouse gonadal tissues. In addition, *TAF4B*-null young mice were initially fertile but became infertile after 3 months because of impaired gonocyte proliferation and reduced expression of spermatogonial stem cell markers.^[18]^ Our study evaluated the association between *TAF4B* variations and NOA in a cohort of Han Chinese patients and controls. We identified 11 known SNPs including 6 synonymous variants and 4 nonsynonymous variants. A novel mutation (c.11G>T/p.G4V) located in exon 1 was detected only in patients with NOA. The mutation was not found in ExAC or 1000G (MutationTaster). ThePolyPhen-2 and SIFT analysis indicated that p.G4V mutations probably altered the protein structure. Evolutionary conservation analysis showed that the residue at amino acid 4 was evolutionarily conserved. TAF4B proteins were modeled by the SWiSS MODel software based on the template of the (2p6v.pdb).^[19]^ We speculate that this mutation may result in altered germ cell function and this should be examined in future studies. Interruption of any of these phases can result in the failure of spermatogenesis and give rise to NOA.^[20]^ Further functional studies are needed to elucidate the effect of these mutations on the changes in the protein structure as well as the biological functions of TAF4B.

One limitation of this study was that the patient group was limited to a Han ethnic population with NOA from Northeast China. Furthermore, the sample size was relatively small in this study. Our bioinformatics results will also need to be confirmed by more cases or animal experiments.

## 5 Conclusions

The present study showed that the c.11G>T mutation of the TAF4B gene may be associated with NOA in a Chinese population. Bioinformatics analysis indicated this variation might play an important role in the process of spermatogenesis. Further investigations are required to explore the functional and pathological role of the mutated TAF4B in NOA.

## Author contributions

**Funding acquisition:** Ruixue Wang.

**Investigation:** Qi Xi, Xinyue Zhang.

**Methodology:** Hao Zhang Yuting Jiang.

**Writing – original draft:** Qi Xi, Hao Zhang.

**Writing – review & editing:** Ruizhi Liu, Hongguo Zhang.
